# Musculoskeletal Effects of Altered GH Action

**DOI:** 10.3389/fphys.2022.867921

**Published:** 2022-05-19

**Authors:** Jonathan A. Young, Shouan Zhu, Edward O. List, Silvana Duran-Ortiz, Yosri Slama, Darlene E. Berryman

**Affiliations:** ^1^ Heritage College of Osteopathic Medicine, Ohio University, Athens, OH, United States; ^2^ Ohio Musculoskeletal and Neurological Institute, Heritage College of Osteopathic Medicine, Athens, OH, United States; ^3^ Department of Biomedical Sciences, Heritage College of Osteopathic Medicine, Ohio University, Athens, OH, United States; ^4^ Edison Biotechnology Institute, Ohio University, Athens, OH, United States

**Keywords:** somatopause, osteoarthritis, sarcopenia, growth hormone, acromegaly, growth hormone deficiency (GHD)

## Abstract

Growth hormone (GH) is a peptide hormone that can signal directly through its receptor or indirectly through insulin-like growth factor 1 (IGF-1) stimulation. GH draws its name from its anabolic effects on muscle and bone but also has distinct metabolic effects in multiple tissues. In addition to its metabolic and musculoskeletal effects, GH is closely associated with aging, with levels declining as individuals age but GH action negatively correlating with lifespan. GH’s effects have been studied in human conditions of GH alteration, such as acromegaly and Laron syndrome, and GH therapies have been suggested to combat aging-related musculoskeletal diseases, in part, because of the decline in GH levels with advanced age. While clinical data are inconclusive, animal models have been indispensable in understanding the underlying molecular mechanisms of GH action. This review will provide a brief overview of the musculoskeletal effects of GH, focusing on clinical and animal models.

## 1 Introduction

Growth hormone (GH) is a peptide hormone commonly known for its role in development and bone and muscle growth. GH is synthesized by somatotrophs in the anterior pituitary, and its secretion is regulated mainly by two hypothalamic hormones, growth hormone releasing hormone (GHRH) and somatostatin, with GHRH increasing and somatostatin decreasing GH secretion, as well as by diet, exercise, stress, and other factors (Caputo et al., 2021). Once secreted, GH circulates and binds to the pre-dimerized GH receptor (GHR) (Brooks and Waters, 2010) on target cells. Receptor binding results in a signaling cascade primarily through Janus Kinase 2 (JAK2) and STAT5. However, other pathways, such as mitogen-activated protein kinases (MAPK) and phosphoinositide 3-kinase (PI3K) pathway (Brooks and Waters, 2010), can also be activated by GH. As for function, GH not only promotes linear postnatal growth and bone growth but also influences metabolism of lipids, carbohydrates, nitrogen, and minerals. Among many favorable tissue-dependent actions, GH is well established to increase muscle mass, reduce adipose tissue through lipolysis, and augment gluconeogenesis in the liver (Olarescu et al., 2000). A notable unwanted action of GH is its ability to inhibit insulin action also known as its diabetogenic activity (Houssay and Biasiotti, 1931).

Importantly, GHR activation stimulates expression of another potent hormone, insulin-like growth factor 1 (IGF-1) in target tissues (Hellstrom et al., 2017). Because of this, GH’s effects on tissues can be either direct, indirect *via* IGF-1, or both direct and indirect (Kopchick and Andry, 2000). For example, GH has been reported to be responsible for ∼14% of longitudinal growth, IGF-1 is responsible for ∼35% (Lupu et al., 2001), and the combined effect of GH and IGF-1 accounts for 34% of growth (and 17% of total mouse growth regulated by other factors). Thus, both GH and IGF-1 have independent and synergistic effects depending on the tissue (Olarescu et al., 2000). IGF-1 shares many intracellular signaling pathways with GH, also activating MAPK and PI3k through insulin receptor substrate 1 (IRS1), and crosstalk between the two pathways has been reported in beta cells (Ma et al., 2011), highlighting the complexity of GH action on target tissues such as muscle and bone.

Although pituitary-derived endocrine GH is a key driver of the musculoskeletal effects of GH, locally produced autocrine/paracrine GH, also known as extrapituitary GH, also plays a major role in the growth and development of muscle and bone (Harvey, 2010). Evidence for the local production of GH is the increased (relative to serum) concentration of GH in the cartilage and synovial fluid of joints (Isaksson et al., 1991; Denko and Malemud, 2005), as well as in skeletal muscle (Kyle et al., 1981; Costa et al., 1993). Autocrine/paracrine GH has also been shown to be associated with muscle cell proliferation and myotube differentiation (Segard et al., 2003).

Extremes in GH action, both elevated and decreased, result in dramatic and distinct clinical conditions that have elucidated the role of this hormone on the musculoskeletal system. Acromegaly is a condition, usually resulting from a pituitary adenoma, that causes GH production in excess of normal physiological levels. The excess GH secretion leads to overproduction of IGF-1 and results in a multisystem disease characterized by somatic overgrowth and disfigurement, multiple comorbidities, and increased mortality (Melmed, 2009). Acromegaly is treated surgically to remove the tumor and/or pharmacologically with somatostatin analogues or the GH receptor antagonist, pegvisomant (Melmed, 2009). Decreased GH action takes two main clinical forms: GH deficiency (GHD), which has myriad causes, and Laron Syndrome (LS), which arises from GH receptor mutations (Laron and Werner, 2021). While the symptoms of GH deficiency vary due to etiology (Aguiar-Oliveira and Bartke, 2019), LS consistently causes short stature, improved glucose metabolism (Guevara-Aguirre et al., 2021) with respect to family members without LS, as well as a reduction in diagnosed malignancies observed in the control group (Guevara-Aguirre et al., 2011). Although GHD and LS may share many symptoms, their treatments differ. GHD is treated using GH replacement therapy with recombinant GH (Aguiar-Oliveira and Bartke, 2019), but in LS, GH replacement therapy is ineffective due to GH resistance, so recombinant IGF-1 is the only option to increase IGF-1 in these individuals (Guevara-Aguirre et al., 2021). All these conditions can result in skeletal and muscular changes that will be discussed briefly below.

Allowing more invasive measurements, various mouse lines have been developed (Table 1) to study the impact of GH action on the entire organism, select tissues or at specific timepoints. For example, bovine GH (bGH) transgenic mice (Knapp et al., 1994) have excess (superphysiological) GH action throughout their life, serving as a model for pediatric acromegaly or gigantism. As might be expected based on the known functions of GH, these mice are giant and lean. Despite their favorable body composition, bGH mice are insulin resistant (Dominici et al., 1999) due to the diabetogenic actions of GH and have a decreased lifespan (Knapp et al., 1994). Likewise, GHR knockout (GHRKO) mice are a model of LS. GHRKO mice have decreased body length and weight, increased insulin sensitivity, and markedly increased lifespan (Coschigano et al., 2003). As for GHD, mice with congenital GH deficiency (GH knockout or −/− mice) (List et al., 2019) and mice with adult GHD *via* GHR disruption starting at 6 months of age (6mGHRKO) (Duran-Ortiz et al., 2021b) or induced somatotroph destruction (Luque et al., 2011; Cordoba-Chacon et al., 2014; Poudel et al., 2021) have recently been developed. Many other mouse lines have also been created that provide insight into GH’s action although they lack clinical correlates. For example, GH signaling disruption in specific tissues such as muscle-specific (Mavalli et al., 2010; Vijayakumar et al., 2012; List et al., 2015), bone-specific (Liu et al., 2016) and liver-specific GHR knockouts have been characterized. This review will mainly summarize the musculoskeletal effects in bGH and GHRKO mice because they have each been more extensively studied.

**TABLE 1:** Summary of the musculoskeletal phenotypes of mouse models with altered GH action.

Mouse line	Body size	Muscle weight	Muscle structure	Muscle strength	Glucose metabolism	Bone phenotype
bGH Transgenic	Increased	Increased proportional to body weight	Shift towards Type I	No change	Insulin resistant	—
GHRKO	Decreased	Decreased proportional to body weight	Shift towards Type II or no change	Increased relative to weight	Improved insulin sensitivity	Decreased trabecular bone volume, cortical bone thickness, BMD, BMC
Muscle-specific GHRKO	Mildly decreased BW in males, mildly increased BW in females	Decreased lean mass in males, mildly increased lean mass in females	—	No change	Improved insulin sensitivity	—
Bone-specific GHRKO	No change	—	—	—	—	Decreased bone formation, trabecular area, cortical area
Liver-specific GHRKO	Decreased at young ages, increased BW at older ages	Decreased lean mass, no change in muscle mass	—	Increased grip strength	—	—
Adult (6 month) GHRKO	Decreased BW at older ages	Decreased lean mass, Decreased quadriceps mass in males	—	—	Improved insulin sensitivity	Thinning of cortex due to increased marrow cavity, decreased lacunar number, increased lacunar volume, increased bone marrow adiposity
AOiGHD	No Change	Decreased lean mass	—	—	Improved insulin sensitivity in males	Increased cartilage degeneration and osteophyte formation in both sexes, increased synovium thickness in males

Important to the aging field, the levels of both GH and IGF-1 decline with advancing age in most mammalian species, referred to as somatopause. This age-related decline in both hormones, along with their potent anabolic effects in the musculoskeletal system, has sparked interest in the use of recombinant GH as an anti-aging drug (Lieberman and Hoffman, 1997). However, longevity data from both humans and mice challenge this notion. That is, mice with reduction or absence in GH action have a robust and reproducible increase in lifespan (Duran-Ortiz et al., 2021a), suggesting an advantage to somatopause on lifespan. While data from humans is insufficient to draw firm conclusions, individuals with LS have no decrease in lifespan and some cohorts of isolated GH deficiency (e.g. Brazilian Itabaianinha cohort) have attained extreme longevity despite representing a relative minor proportion of the population, suggesting that the findings in rodents are relevant to humans (Junnila et al., 2013). Despite the impact on lifespan, the benefits of GH supplementation on the aging musculoskeletal system have not been fully explored. Thus, this brief review will provide a summary of the complex effects of GH on the musculoskeletal system, focusing on extremes in GH action and on age-related conditions from clinical cohorts as well as *in vivo* data using animal models.

## 2 GH in Muscle

Skeletal muscle is a primary target for GH (and IGF-1) with growth-promoting effects. GH also has metabolic effects, with a well-documented ability to influence insulin-stimulated glucose uptake in skeletal muscle (Moller and Jorgensen, 2009). Below summarizes the impact of extremes in GH action (acromegaly and GH deficiency) on body composition and skeletal muscle structure and function from clinical studies as well as the role of GH in age-associated changes in skeletal muscle. Data from animal studies provides additional metabolic and physiological features of GH in muscle.

### 2.1 Clinical Data

#### 2.1.1 Extremes in GH Action

##### 2.1.1.1 Acromegaly

Patients with acromegaly have increases in lean mass and total body water (mainly extracellular water) (Bengtsson et al., 1989; Freda et al., 2009; Fuchtbauer et al., 2017). Despite the increase in lean mass, acromegaly is commonly believed to be associated with myopathy (characterized by muscle weakness and pain) and reduced muscle endurance (Mastaglia et al., 1970; Lopes et al., 2016). This acromegaly-associated myopathy can be a debilitating co-morbidity and is often considered a major contributor to the reduced quality of life reported for these patients (Miller et al., 2008). In terms of muscle function, cross-sectional studies have shown a decrease in hand grip strength, decreased peak torque, reduced maximal repetition in knee extension and flexion along with lateral instability (Lopes et al., 2016; Homem et al., 2017). However, a recent longitudinal study reports contrary results with a normal to modest increase in strength although confirmed the presence of reduced grip strength with active acromegaly (Fuchtbauer et al., 2017). While studies are limited, there are several structural changes in skeletal muscle with active acromegaly that are thought to contribute to the muscle dysfunction. That is, hypertrophy is noted in several studies but not all (Freda et al., 2009; Ozturk Gokce et al., 2020). As for muscle fiber type, most studies report hypertrophy of type I fibers but variable findings for type II fibers (Mastaglia et al., 1970; Nagulesparen et al., 1976; Khaleeli et al., 1984). An increase in intramuscular fat content has also been shown with active acromegaly (Reyes-Vidal et al., 2015). The excess GH associated with acromegaly is associated with insulin resistance and impaired insulin-stimulated glucose uptake in skeletal muscle (Moller et al., 1992; Moller and Jorgensen, 2009). Disease management improves but does not completely ameliorate the myopathy. For example, in the longitudinal study described above (Fuchtbauer et al., 2017), grip strength improves after disease remission but proximal muscle fatigue increases. Fat infiltration in the muscle also remains in controlled acromegaly (Martel-Duguech et al., 2021). Improvement in insulin resistance in muscle occurs with disease remission but varies depending on treatment modality (Dal et al., 2016).

##### 2.1.1.2 GH Deficiency

Adults with GH deficiency (GHD) have decreased lean mass along with reduced isometric muscle strength (Jorgensen et al., 1989; Cuneo et al., 1991; Johannsson et al., 1997). Muscle endurance and isokinetic muscle strength are either reduced or in the lower range of normal (Cuneo et al., 1991; Johannsson et al., 1997). In general, GH replacement therapy of GHD increases lean body mass through both increasing skeletal muscle mass and tissue hydration (Jorgensen et al., 1989; Salomon et al., 1989; Cuneo et al., 1991; Rutherford et al., 1995; Elbornsson et al., 2013), as also confirmed in a comprehensive analysis of 51 clinical trials (Klefter and Feldt-Rasmussen, 2009). Of note, the increase in hydration of the muscle with GH therapy complicates the interpretation of most studies on GH therapy as methods used do not always differentiate between extracellular water and intracellular mass, which also confounds data presented above for acromegaly. GH replacement therapy in GHD for one year is associated with a 5–10% increase in muscle volume as assessed by either computed tomography or dual-energy X-ray absorptiometry scanning (Jorgensen et al., 1996). In a study that tracked patients for 10 years, GH treatment in patients with GHD confirms an increase muscle strength during the first years of treatment and partially protects from declines in muscle strength and neuromuscular function that occurs with aging (Gotherstrom et al., 2009).

As for other muscle properties, either no change in proportion of fiber types (Whitehead et al., 1989; Bottinelli et al., 1997) or data consistent with a larger proportion of type II fibers (Rutherford et al., 1995) has been reported for GHD, which normalize after GH therapy. Individuals with GHD may also have reduced capacity to restore intramyocellular lipids after aerobic exercise (Loher et al., 2018) although other studies do not show this trend (Christ et al., 2016). With respect to metabolism, untreated GHD is associated with reduced glycogen along with decreased insulin-stimulated glycogen synthase activity in skeletal muscle (Hew et al., 1996; Christopher et al., 1998). These defects persist after two years of GH treatment; that is, GH treatment of GHD leads to continued inhibition of insulin-stimulated glycogen synthase activity, accompanied by a reduced baseline glycogen content, low-to-normal glucose 6 phosphate levels, and high total intracellular glucose concentrations in skeletal muscle (Christopher et al., 1998), a unique combination to induce insulin resistance.

#### 2.1.2 GH Action and Sarcopenia and Dynapenia

Sarcopenia and dynapenia are the age-associated loss of muscle mass and strength, respectively (Clark and Manini, 2008). As noted above, secretion of GH and IGF-1 decline with age such that low levels are detected in individuals over 60 years of age (Clemmons and Van Wyk, 1984; Zadik et al., 1985; Iranmanesh et al., 1991). These changes have sparked interest in using these hormones as therapy to combat age-related changes in muscle. Indeed, patients with sarcopenia have been reported to have lower GH and IGF-1 levels (Bian et al., 2020), with the severity of sarcopenia associated with reduced serum IGF-1 (Jarmusch et al., 2021). GH therapy in older adults has shown positive effects on body composition, with an increase in lean mass and a decrease in fat tissue (Taaffe et al., 1994; Papadakis et al., 1996; Franco et al., 2005). However, increase in muscle mass is not usually related to an improved physical ability or muscle strength (Taaffe et al., 1994; Papadakis et al., 1996; Lange et al., 2002) and has thrown doubt on its use as a strategy to combat sarcopenia or dynapenia. As noted before, the increases in lean mass may reflect increased fluid retention as opposed to lean tissue. Despite these discouraging results, some studies in elderly subjects that combine resistance training with GH therapy show improvements in muscle strength and a change in fiber types (Hennessey et al., 2001). Further recent studies suggest that a subset of patients with sarcopenia may be GH resistant (Ferrari et al., 2021), which may influence response to hormone therapy. Finally, recent data suggests the low IGF-1 in sarcopenia may be pathological to the muscle due to its potent effects on neurons (enhancing neuronal survival, neurite formation and outgrowth in motoneurons) (Jarmusch et al., 2021). Despite the integral role of the GH/IGF axis on muscle metabolism and hypertrophy, the use of GH or IGF-1 for its anti-aging properties remains controversial.

### 2.2 Mouse Data

#### 2.2.1 Mice With Excess GH Action

GH’s role in muscle has been evaluated using bGH transgenic mice. bGH mice have increased muscle mass compared to controls, but muscle mass relative to body mass is unchanged (Schuenke et al., 2008). Muscle structure is changed, with a shift towards type I fibers, and an increased cross-sectional area across fiber types (Schuenke et al., 2008). Despite their increased muscle size, male bGH mice have similar grip strength to controls, indicating a less efficient muscle (Wolf et al., 1995). Increased muscle atrophy signals are also observed, with 5 month old bGH mice having increased circulating myostatin as well as increased MuRF1 expression in the gastrocnemius but not the soleus (Consitt et al., 2017). Skeletal muscle of bGH-transgenic mice has impaired insulin signaling at several levels, including 1) reduced insulin receptor abundance, 2) reduced insulin receptor tyrosine phosphorylation, 3) reduced IRS-1 tyrosine phosphorylation, and 4) defective activation of PI3K by insulin (that is, the association of IRS-1 with the p85 subunit is increased more 375% under basal conditions due to excess GH signaling which prevents its use for insulin signaling) (Dominici et al., 1999).

*In vitro*, cultured mouse limb myoblasts from wild type mice treated with GH show increases in myofiber size by promoting fusion of satellite-like myoblasts to nascent myotubes (Sotiropoulos et al., 2006). However, GH treatment of these limb myoblasts has no effect on size, proliferation, or differentiation of myoblast precursor cells indicating that GH plays a role in muscle cell fusion, rather than stimulating hyperplasia or hypertrophy of myoblast precursor cells (Sotiropoulos et al., 2006).

To delineate the effects of GH vs. endocrine IGF-1 action, several mouse lines that have a liver-specific ablation of the GHR have been generated, as most endocrine IGF-1 is produced in the liver in response to GH. These mice have intact GHR in all the tissues of the body, except for the liver; as a result, they present high GH, but low IGF-1 levels in the serum, having a form of extrahepatic acromegaly. These liver specific knockout animals have decreased lean mass but no change in quadriceps muscle mass and increased grip strength, indicating that high GH in the absence of high circulating IGF-1 may have a positive effect on muscle strength (List et al., 2014).

#### 2.2.2 Mice With Reduced GH Action

Decreased GH action in muscle has been examined using GHRKO mice. While these mice have normal numbers of fibrils, myofiber size is reduced, resulting in an overall decrease in muscle mass (Sotiropoulos et al., 2006; Schuenke et al., 2008). Muscle fiber type has also been evaluated in these mice with mixed results. Sotiropoulos et al. (Sotiropoulos et al., 2006) report that soleus and tibialis anterior muscles from 2 month old GHRKO mice have a higher proportion of type II vs. type I fiber type compared to control mice. In contrast, Schuenke et al. (Schuenke et al., 2008) later reports no such change in fiber types from soleus, plantaris, and gastrocnemius at 4 months of age. These results suggest that the proportion of fiber types may depend on mouse age, strain, sex, and perhaps different muscle groups used. When muscle function is assessed using grip strength (normalized to body weight), 7 month old GHRKO mice show improvement compared to controls (Lozier et al., 2018). An aspect of the sex-specific effects of GH alterations has been further evaluated using orchidectomized GHRKO and WT mice and treatment with testosterone during late puberty (Venken et al., 2007). Testosterone treatment stimulates a similar increase in muscle mass in both GHRKO and WT mice indicating that androgens and GH stimulate muscle growth *via* distinct mechanisms (Venken et al., 2007). In terms of insulin metabolism, the muscles of GHRKO mice have increased insulin receptor expression, increased insulin stimulated p85 phosphorylation, increased insulin stimulated phosphorylated AKT1 and AKT2 phosphorylation, and increased total GLUT4 protein concentration (Bonkowski et al., 2009). Interestingly, two separate laboratories have demonstrated that when GHR is disrupted selectively in muscle, whole body insulin sensitivity is enhanced (Vijayakumar et al., 2012; List et al., 2015), with no reported change in muscle strength (List et al., 2015). In accordance with improved insulin sensitivity seen in muscle-specific GHRKO mice, these mice have a modest increase in lifespan (List et al., 2015).

Taken together, studies in humans and in numerous mouse models of altered GH action indicate that GH signaling is positively associated with muscle mass, but the increase in mass seen with GH excess does not confer increased muscle strength, possibly due to the altered structure of the muscle. Decreased GH signaling leads to decreased muscle mass, but the muscles appear to be relatively stronger despite inconsistent changes to muscle structure.

## 3 GH in Bone/Joints

GH (and IGF-1) also have robust anabolic effects on bone, stimulating osteoblast differentiation, linear bone growth, and increased BMD, among others. Mouse lines with altered GH/IGF-1 axis have been used to assess the role of GH on bone acquisition and metabolism. Overall, excess GH action results in augmented skeletal growth (D'Ercole, 1993), while reduction in the action of the GH/IGF-1 axis results in mice that, despite having normal body weight at birth, show a postnatal reduced skeletal acquisition compared to controls (Duran-Ortiz et al., 2021a). Below we summarize the skeletal phenotypes of mouse models with excess or reduced GH action.

### 3.1 Clinical Data

#### 3.1.1 From Extremes in GH Action

##### 3.1.1.1 Acromegaly

GH excess in acromegaly leads to increased bone turnover, as evidenced by increases in biochemical markers for both bone formation and resorption (Aloia et al., 1972; Lepszy et al., 1976; Halse et al., 1981; Halse and Gordeladze, 1981; de la Piedra et al., 1988; Ezzat et al., 1993; Kotzmann et al., 1993; Kayath and Vieira, 1997; Bolanowski et al., 2006). However, this increase in bone turnover does not correlate well with changes of bone mineral density (BMD) at the local level. Decreased BMD in lumbar spine and femoral neck has been reported in various studies (Aloia et al., 1972; Riggs et al., 1972; Halse et al., 1981; Seeman et al., 1982; Diamond et al., 1989; Kayath and Vieira, 1997; Lesse et al., 1998; Longobardi et al., 1998; Chiodini et al., 2001; Bolanowski et al., 2006), suggesting a higher rate of bone resorption versus formation. Consequently, a higher incidence of radiographical vertebral deformities and fractures has been reported in acromegaly (42.0%) compared to control subjects (3.8%) (Claessen et al., 2013). This suggests that acromegaly is linked to an increased risk of osteoporotic vertebral fracture. Whereas an opposite effect–increases in BMD–is observed in the forearm of patients with acromegaly (Seeman et al., 1982; Diamond et al., 1989). This can partially be explained by the differential responses of trabecular (such as in lumbar spine) and cortical bone (such as in forearm) to excess GH. Despite the strong anabolic effect of GH on bone, the net BMD gain/loss is likely a result of complex interactions of sex steroids with GH/IGF-1 axis, as reviewed elsewhere (Birzniece and Ho, 2017).

Joint manifestations are one of the most common clinical complications in patients with acromegaly. Either axial or peripheral arthropathy has been reported in more than 50% of patients (Layton et al., 1988). Typical radiographic osteoarthritic changes including joint space narrowing, osteophytosis, subchondral bony sclerosis, and cysts formation can be seen in some but not all patients with acromegaly (Layton et al., 1988; Colao et al., 2005). Some argue that those pathological changes might not be indicative of an osteoarthritis (OA) diagnosis as patients with acromegaly often times also have radiographic signs in the hand and spine joints unlike those commonly seen in OA (Tornero et al., 1990). Nevertheless, joint manifestation associated pain is one of the most common complications that greatly affects quality of life in long-standing acromegaly (Miller et al., 2008; Kropf et al., 2013).

##### 3.1.1.2 GH Deficiency (GHD)

Decreased BMD has been consistently reported in patients with GH deficiency (GHD), either isolated or combined with other pituitary hormone deficiencies (Kaufman et al., 1992; Ohlsson et al., 1998; Wuster et al., 2001; Doga et al., 2005). The degree of bone loss is dependent on the sites, the duration and age of GHD onset, and the age of patients. Current evidence suggests that comparing with trabecular bone, cortical bone is more targeted by GHD (Johansson et al., 1992; Wuster et al., 2001). Consistently, the risk of nonvertebral fracture is increased approximately 3-fold in patients with GHD and the fractures are frequently localized to the radius (Johansson et al., 1992; Rosen et al., 1997; Wuster et al., 2001), a site rich in cortical bone. Additionally, patients with childhood-onset GHD are smaller and have a greater decrease of bone mass than patients with adult-onset GHD (Attanasio et al., 1997; Lissett and Shalet, 2002). This is thought to be due to missing effect from GH on reaching the peak bone mass during puberty (Bonjour et al., 1991). In contrast, the degree of bone loss in adult-onset GHD correlates with the age of patients and the duration and severity of the disease (Rosen et al., 1993; Holmes et al., 1994; Toogood et al., 1997; Colao et al., 1999; Murray et al., 2004; Fee and Bu, 2007). Conversely, when long-term recombinant GH therapy is used to treat patients with GHD, there is a resulting increase in BMD, with no change observed in trabecular bone score (Vanuga et al., 2021). Collectively, these studies demonstrated that GHD can contribute to bone loss and osteoporosis.

Not much is known about GHD on OA development. One comparative study has found that the prevalence of radiographic OA is lower in elderly patients with GHD than a normal population of elderly people (Bagge et al., 1993). Recently, a polymorphism of human GH receptor (GHR), the genomic deletion of exon 3 (d3GHR), was identified to be associated with increased growth velocity in children with GH deficiency (Urbanek et al., 1993; Dos Santos et al., 2004). This polymorphism enhances GH’s growth-promoting effects although GHR binding is not altered (Urbanek et al., 1993; Dos Santos et al., 2004). Interestingly, patients with d3GHR mutation have increased prevalence of OA, especially in hip joint, while both BMD and rate of (non)vertebral fractures are not significantly altered (Wassenaar et al., 2009; Claessen et al., 2014).

#### 3.1.2 GH/IGF-1 Action and Age-Related Osteoporosis and Osteoarthritis

The level of GH and IGF1 decrease with aging (Clemmons and Van Wyk, 1984; Zadik et al., 1985; Iranmanesh et al., 1991), correlating with the increased risk of osteoporosis and fragility fracture in elderly population. A positive relationship between BMD and concentration of IGF-1 and IGF binding protein 3 (IGFBP-3) in serum is observed in healthy men (Johansson et al., 1994), and low serum IGF-1 level is associated with increased risk of hip and vertebral fractures (Ohlsson et al., 2011). However, the age-dependent decrease of GH and IGF-1 does not seem to always correlate with the reduction of BMD in humans. For example, in postmenopausal women with osteoporosis, serum IGF-1 does not differ from that in postmenopausal women without osteoporosis (Bennett et al., 1984). This suggests that aging associated skeletal homeostasis is likely a multifactorial process mediated by sex hormones, GH/IGF-1 axis, and others. Not surprisingly, the administration of GH or IGF-1 for treating osteoporosis in clinical trials has yielded mixed results. For example, in a randomized placebo-controlled trial in postmenopausal women with up to three years of follow-up, GH therapy results in 14% increase of BMD (Landin-Wilhelmsen et al., 2003), a result that was not reproduced in earlier trial (Saaf et al., 1999). The role of aging dependent decline of GH/IGF-1 on OA development has remained largely unknown, though one study showed that there was no direct link between either GH or IGF-1 serum level and OA (Lis, 2008).

### 3.2 Mouse Data

#### 3.2.1 Mice With Excess GH Action

Mouse lines with excess GH, such as mice that overexpress human GHRH, human GH and bGH, show increased bone and body size (Wolf et al., 1991; D'Ercole, 1993; Jensen et al., 2021). Note that human GH binds to the prolactin receptor in addition to the GH receptor, while bGH only binds to the GH receptor. Therefore, bGH mice are a better model to study the specific effects of augmented GHR activation. bGH mice have increased bone length but compromised bone architecture and BMD with reduced trabecular bone volume fraction and thickness (Lim et al., 2015). Also, cortical tissue perimeter is increased in bGH mice, but cortical thickness is reduced. In lumbar vertebra, bGH mice show similar trabecular BMD but reduced trabecular thickness relative to controls while cortical BMD and thickness are significantly reduced in bGH mice. Importantly, at 5 months of age, bone turnover is increased in favor of bone resorption in at least bGH tibia (Lim et al., 2015). Pathohistological analysis of the knee joints from bGH mice at 6 months of age reveal that the mice have loss of articular cartilage zonal structure, presence of hypertrophic chondrocytes, and thickening of synovial lining tissue and pannus, suggesting osteoarthritic degeneration happens at an early age with GH overproduction (Eckstein et al., 2002; Eckstein et al., 2004). Similar cartilage degeneration is also seen in the hip joints of bGH mice (Munoz-Guerra et al., 2004).

The effects of GH action in bone are age- and sex-dependent in terms of BMD, cortical area, and bone minerals. For example, in bGH transgenic mice, only males have increased cortical cross-sectional area, yet females have increased trabecular density, femoral bone density, and trabecular bone volume fraction. However, the increased trabecular density in females was limited to younger ages between 6 and 12 weeks. Both sexes have decreased cortical density and mineralized tissue matrix density. It is apparent that in adulthood, excess GH action has a negative effect on bone structure (Eckstein et al., 2002; Eckstein et al., 2004). As noted previously, mice with a liver-specific GHR knockout have high circulating GH and low circulating IGF-1. Interestingly, different studies have found conflicting results for the body length of these mice, with two studies showing no difference (Yakar et al., 1999; Fan et al., 2009) and one study displaying significant decrease in body length (List et al., 2014) of the liver-specific GHR knockout mice compared to controls. These mice have impaired BMD with impaired cortical bone acquisition, microarchitecture, trabecular bone volume, and strength. With the exception of trabecular bone volume, these deficiencies are rescued when hepatic IGF-1 production is normalized by crossing these mice with hepatic IGF-1 transgenic mice (Liu et al., 2018). These results implicate IGF-1 as a major contributor to skeletal growth and structure.

As always when studying GH, it is difficult to tease out direct effects of GH from those mediated through IGF-1. In an *in vitro* experiment using osteoblasts from mice with an osteoblast-specific IGF-1R deletion, GH was shown to activate JAK2 and STAT5 normally. Other pathways had differing responses, as ERK activation by GH was normal in the absence of IGF-1R, while Akt activation was blunted.

#### 3.2.2 Mice With Reduced GH Action

Germline reduction in GH action as seen in GHRKO mice results in mice with ∼50–60% decreased body size (List et al., 2019). Besides reduced longitudinal growth, 3 month old GHRKO mice also show decreased trabecular bone volume, as well as reduced cortical bone total cross-sectional area, bone area, cortical bone thickness, periosteal/endosteal circumference and reduced BMD and bone mineral content (BMC) (Sjogren et al., 2000). Furthermore, reduced skeletal growth in GHRKO mice has been associated with premature growth plate closure and reduced chondrocyte proliferation, bone turnover and periosteal bone apposition (Sims et al., 2000). To study the effects of reduced GH action postnatally, a mouse line with disrupted GHR at 6 months of age (6mGHRKO) was recently reported (Duran-Ortiz et al., 2021b; Dixit et al., 2021). Disrupting GHR globally at an adult age results in more slender bones, expansion of the marrow cavity, reduced osteocyte lacunar number, and increases in lacunar volume and loss of canalicular connectivity (Dixit et al., 2021). However, mineral/matrix ratio is not altered. Collectively, these studies show that germline and postnatal reduction of GH compromises morphology and development of the bones.

Moreover, one recent study examining an adult-onset isolated GH deficiency (AOiGHD) model found that reduction of GH during adulthood leads to increased cartilage degeneration and osteophyte formation in both male and female, while synovium thickening only in male (Poudel et al., 2021). Although not in mice, chronic GH/IGF-1 deficiency in a dwarf rat model (dw/dw) causes an increased severity of articular cartilage lesion of OA without formation of osteophytes and subchondral sclerosis. Interestingly, the cartilage lesion is ameliorated by a life-long repletion of GH (Ekenstedt et al., 2006). These results suggest a beneficial effect of reduced GH action on joint health, which is inconsistent from the aforementioned, pronounced OA phenotype in the bGH mice. This discrepancy is likely due to multiple factors including different genetic modified animal models, different species, and germline versus inducible deletion of GH.

To study the effects of GH action specifically on the bone, a mouse line with the GHR disrupted only in bone was generated, which results in bones that are resistant to GH but responsive to IGF-1. These mice, called dentin matrix protein (DMP)-1 GHRKO (DMP-GHRKO) mice (Liu et al., 2016), do not have any significant change in body weight, composition, and growth, although they show differences in bone acquisition. That is, DMP-GHRKO mice have decreased bone formation, mineral deposition rate, reduced cortical and trabecular areas, and increased and decreased number of osteoclasts and osteoblasts, respectively compared to controls (Liu et al., 2016). Altogether, these results show that germline reduction of GH action results in impaired skeletal growth and decreased bone mineral density.

## 4 Experimental Variant of Human GH

While this review has focused on “GH”, it is important to remember that the human GH gene family is made up of five genes that share similar structure. These five genes include, GH-N (the main focus of this review), GH-V (also called placental GH), chorionic somatomammotropin hormone 1 (also called placental lactogen 1), chorionic somatomammotropin hormone 2 (also called placental lactogen 2), and a pseudogene called chorionic somatomammotropin-like hormone. While GH-N is produced mainly in the pituitary, all other members of the GH gene family are produced in the placenta. GH-V is 93% identical to GH-N at the amino acid level, and the 22K isoform, which is the most abundant isoform of GH-V, has been shown to promote growth and mediate maternal insulin resistance during pregnancy (Miller and Eberhardt, 1983; Solomon et al., 2006). Another isoform, a 20K GH-V isoform, is expressed at low levels (Boguszewski et al., 1998); however, two separate laboratories indicate that the 20K GH-V isoform has potent effects when given at therapeutic levels. Specifically, 20K GH-V stimulates IGF-1, increases longitudinal bone growth, increases muscle mass, and reduces fat mass when injected into mice and rats (Vickers et al., 2009; List et al., 2020). Importantly, while 20K GH-V appears to have full anabolic activity, it lacks diabetogenic and lactogenic activities found in human GH-N (Vickers et al., 2009; List et al., 2020). The clinical implications of a GH that lacks diabetogenic and lactogenic activities is attractive since both of these activities are generally associated with negative health consequences. While still very preliminary and only in rodents, these data suggest that 20K GH-V may represent improvements to current GH therapies for bone or muscle-related maladies.

## 5 Conclusion

Growth hormone has its most notable effects on the musculoskeletal system, as these effects lend GH its name. Overall, GH acts directly and indirectly through IGF-1 to increase bone and muscle mass, but the increase in mass does not necessarily result in increased strength in either tissue. The lack of increased strength may be explained by changes in fiber type in muscle and decreased BMD in some types of bone. GH deficiency causes decreased bone and muscle mass and strength, and GH treatment of GHD increases bone and muscle mass and muscle strength. Animal models of increased (bGH mice) and decreased (GHRKO mice) GH action mirror the results seen in their respective clinical populations.

Many questions remain unanswered. First, what is the role of GH versus IGF-1? A key aspect of GH action is the distinction between its direct and IGF-1 mediated effects, but in clinical populations (with the exception of rare conditions like LS), high circulating GH is coupled with high circulating IGF-1 or vice versa. A mouse model that decouples high GH from high IGF-1 (Liver specific GHR knockout mice, which have high GH but low IGF-1) shows a distinct muscle phenotype with increased grip strength. Likewise, IGF-1 transgenic mice, with high IGF-1 but low GH, have increased body weight but no increase in skeletal growth (Mathews et al., 1988). A previous review has summarized the distinct and overlapping effects of GH and IGF-1 on bone (Yakar and Isaksson, 2016), highlighting the complexity of this issue.

Second, does GH have potential utility for treating age-related conditions? GH and IGF-1 have significant anabolic effects in both skeletal muscle and bone, making them of high interest as an anti-aging therapy for disorders in these tissues (Bartke, 2019). To date, data are sparse for the specific benefits of GH therapy for both sarcopenia and OA and inconsistent for other conditions, such as osteoporosis. Further, GH resistance, treatment regimen/duration, age of treatment and sex are all important variables that have not been appropriately assessed. However, there are negative metabolic consequences of using GH in older adults. That is, as GH promotes insulin resistance, promoting diabetes. Further, both GH and IGF-1 appear to contribute to the development, progression, therapy resistance and metastases of multiple human cancers expressing GHRs (Basu and Kopchick, 2019). Controlled exposure to GH or the use of GH variants, which have the anabolic effect on bone and muscle but lack some of the diabetogenic action (List et al., 2020), are possible modes of treatment. Thus, the intricate links between GH, the musculoskeletal system, metabolism, and lifespan have room for further investigation.
